# Anesthesia-Induced Developmental Neurodegeneration: The Role of Neuronal Organelles

**DOI:** 10.3389/fneur.2012.00141

**Published:** 2012-10-11

**Authors:** Vesna Jevtovic-Todorovic, A. Boscolo, V. Sanchez, N. Lunardi

**Affiliations:** ^1^Department of Anesthesiology, University of Virginia Health SystemCharlottesville, VA, USA; ^2^Neuroscience Graduate Program, University of VirginiaCharlottesville, VA, USA; ^3^Department of Anesthesiology and Pharmacology, University of PaduaPadua, Italy

**Keywords:** immature brain, cognitive impairment, general anesthetics, antiepileptics, mitochondria, endoplasmic reticulum, autophagy, neuroapoptosis

## Abstract

Exposure to general anesthetics (GAs) and antiepileptics during critical stages of brain development causes significant neurotoxicity to immature neurons. Many animal, and emerging human studies have shown long-term functional sequelae manifested as behavioral deficits and cognitive impairments. Since GAs and antiepileptic drugs are a necessity, current research is focused on deciphering the mechanisms responsible for anesthesia-induced developmental neurotoxicity so that protective strategies can be devised. These agents promote massive and wide-spread neuroapoptosis that is caused by the impairment of integrity and function of neuronal organelles. Mitochondria and endoplasmic reticulum are particularly vulnerable. By promoting significant release of intracellular calcium from the endoplasmic reticulum, anesthetics cause an increase in mitochondrial calcium load resulting in the loss of their integrity, release of pro-apoptotic factors, functional impairment of ATP synthesis, and enhanced accumulation of reactive oxygen species. The possibility that GAs may have direct damaging effects on mitochondria, resulting in the impairment of their morphogenesis, also has been proposed. This review will present evidence that neuronal organelles are critical and early targets of anesthesia-induced developmental neurotoxicity.

## Introduction

Rapid advances in pediatric anesthesiology and neurology have resulted in numerous exposures of the children to a variety of psychotropic agents that modulate neuronal activity. Although our ability to take care of very sick children, including very premature infants, has improved immensely, it remains to be determined whether and how early exposure to general anesthetics (GAs) affects the development of a very young brain.

To begin to address this issue, we should get some sense as to how GAs modulate neuronal activity. The mechanisms of action of clinically used GAs are not fully understood. However, it is becoming increasingly evident that there are specific cellular targets through which GAs act (Franks, 2008). Enhancement of inhibitory and/or inhibition of excitatory synaptic transmission have been reported. Many intravenous anesthetics, including barbiturates, benzodiazepines, propofol, and etomidate (Hirota et al., 1998; Franks, 2008), as well as inhalational volatile anesthetics, such as isoflurane, sevoflurane, desflurane, and halothane (Pearce, 2000; Nishikawa and Harrison, 2003), promote inhibitory neurotransmission by enhancing γ-amino-butyric acid type A receptor (GABA_A_)-mediated currents. Intravenous anesthetic ketamine (Lodge and Anis, 1982) and the inhalational anesthetics nitrous oxide and xenon (Jevtovic-Todorovic et al., 1998; Franks, 2008) inhibit excitatory neurotransmission by blocking *N*-methyl-d-aspartate (NMDA) receptors, a subtype of glutamate receptors.

Our brain is not fully developed at birth and extensive synaptogenesis that starts during the last trimester of *in utero* life continues during the first few years of post-natal life (Dobbing and Sands, 1979; Brown et al., 1997). Since glutamate and GABA regulate all key elements of synaptogenesis, we begin to question how anesthesia-induced disturbance of the fine balance between glutamatergic and GABAergic neurotransmission during a crucial stage of brain growth may affect development of neuronal networks. Could it be that early exposure to anesthesia may constitute a generic signal for developing neurons to “commit suicide,” i.e., die by apoptosis? After all, young neurons that are not successful in making meaningful connections are considered superfluous and are destined to die by apoptosis, a natural process during early stages of normal brain development (i.e., neurogenesis) resulting in the removal of 50% or more cells (White et al., 1998). But what is the natural degree of neuronal death during later stage of brain development (i.e., synaptogenesis)? Apoptosis during normal synaptogenesis is a tightly controlled phenomenon, resulting in the removal of only a small percentage of neurons (Jevtovic-Todorovic et al., 2003). This process is disturbed by the exposure to GAs thus “pushing” many neurons into the redundant category destined to die. Recently published findings (Jevtovic-Todorovic et al., 2003; Young et al., 2005; Slikker et al., 2007; Rizzi et al., 2008; Loepke et al., 2009) suggest that common GAs do indeed cause apoptotic degeneration of developing neurons in various mammalian species. The vulnerability to anesthesia-induced neuroapoptosis coincides with synaptogenesis, although vulnerability observed during early and late stages of synaptogenesis may vary (Yon et al., 2005; Rizzi et al., 2008). In some cases the anesthetic-caused neurodegeneration in rodents declines or may not be detected during second/third post-natal weeks (Briner et al., 2010), a period of the rapid phase of synaptogenesis in rat cortex (O’Callaghan, 1992; Schachtele et al., 2011) despite significant disturbance in synapse formation.

Aside from prominent caspase-3 staining at the light microscopic level, the ultrastructural examination shows the clumping of chromatin, disruption of the nuclear membrane, and the formation of apoptotic bodies (Jevtovic-Todorovic et al., 2003). Numerous animal (Jevtovic-Todorovic et al., 2003; Fredriksson et al., 2004, 2007; Li et al., 2007; Paule et al., 2011) and recently emerging human studies (Hack et al., 2005; Hintz et al., 2005; Chorne et al., 2007; Rees et al., 2007; Sun et al., 2008; Kalkman et al., 2009; Wilder et al., 2009; Sprung et al., 2012) suggest that early exposure to GA disturbs the development of cognition, motivation, and attention. The functional link between massive and wide-spread neuroapoptosis and behavioral development remains to be determined.

Since exposure to GAs often cannot be avoided when a child’s well-being is in danger, a considerable effort has been made in recent years to elucidate the mechanisms of anesthesia-induced developmental neuroapoptosis. In this manuscript, presently available evidence regarding the role of neuronal organelles, mitochondria, and endoplasmic reticuli (ER) in particular, in initiating and propagating anesthesia-induced developmental neurotoxicity is reviewed.

## The Role of Mitochondria in Anesthesia-Induced Developmental Neurotoxicity

**Figure 1:**
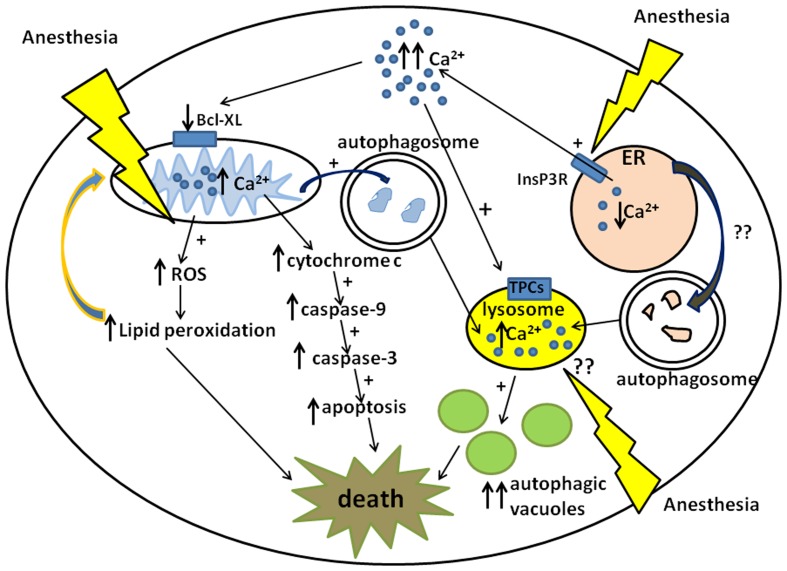
**Schematic diagram of anesthesia-induced pathways of developmental neurodegeneration**. Three proposed pathways are focused on mitochondria, endoplasmic reticuli (ER), and lysosomes: (1) ER-dependent pathway involves anesthesia-induced activation of inositol 1,4,5-trisphosphate receptors (InsP3R) leading to the excessive calcium (Ca^2+^) release and acute elevation of cytosolic Ca^2+^. This causes downregulation of mitochondrial anti-apoptotic protein, bcl-x_L_, which in turn induces cytochrome *c* leak in the cytoplasm. Cytochrome *c* activates mitochondrial apoptotic pathway by activating caspase-9 and -3 leading to DNA fragmentation and neuronal death. (2) Mitochondria-dependent pathway also involves anesthesia-induced up-regulation of reactive oxygen species (ROS) leading to the excessive lipid peroxidation of lipid membranes and damage to neuronal organelles, mitochondria, and ER in particular. Since damaged mitochondrial can become an uncontrollable source of ROS and cytochrome *c* whereas damaged ER can become an uncontrollable source of cytosolic Ca^2+^ they have to be removed by autophagy leading to the excessive formation of autophagosomes and increase in autophagic load. (3) Lysosome-dependent pathway involves lysosome activation via nicotinic acid adenine dinucleotide phosphate (NAADP) gated two-pore channels (TPCs) which control Ca^2+^uptake into the lysosomes. An increase in intralysosomal level of Ca^2+^activates lysosomal activity which in turn promotes lysosomal and autophagosomal fusion, the formation of autophagic vacuoles and neuronal “self-eating.” Although it is proposed that anesthesia causes lysosomal activation indirectly via an increase in cytosolic Ca^2+^from the ER, it remains unclear whether anesthesia has a direct effect on lysosomal activation (via NAADP-gated TPCs in particular).

Very early events involve activation of a mitochondria-dependent apoptotic cascade (Yon et al., 2005, 2006), suggesting that mitochondria may be a vulnerable target. Apoptosis occurs via different biochemical pathways, resulting in activation of effector caspases. The mitochondria-dependent pathway involves the downregulation of anti-apoptotic proteins from the bcl-2 family (e.g., bcl-x_L_), an increase in mitochondrial membrane permeability, followed by an increase in cytochrome *c* release into the cytoplasm which activates caspases-9 and -3, resulting in apoptosis (Figure 1). GAs administered during synaptogenesis activate the mitochondria-dependent cascade within the first 2 h after anesthesia exposure as shown by a significant decrease in protein levels of bcl-x_L_, a rise in cytochrome *c*, and activation of caspase-9 (Yon et al., 2005).

GAs also cause a long-lasting disturbance in mitochondrial morphogenesis. Two weeks postanesthesia exposure, mitochondria were enlarged with deranged, fragmented cristae, and inner membranes, suggesting significant impairment of mitochondrial membrane integrity (Sanchez et al., 2011). Although it is possible that anesthesia-induced mitochondrial enlargement could be due to swelling, it is noteworthy that mitochondrial regeneration in neurons depends on fine dynamics between mitochondrial fusion and fission (Chan, 2006); deranged fusion leads to fragmentation while deranged fission leads to enlargement. We believe that GAs may disturb fine mitochondrial dynamics leaning toward excessive mitochondrial fusion and impaired fission, which may not be well tolerated by immature and functionally busy mammalian neurons. Indeed, impairment of mitochondrial morphogenesis may be, at least in part, the cause of the reported anesthesia neurotoxicity (Ikonomidou et al., 1999, 2000; Yon et al., 2005; Lu et al., 2006; Slikker et al., 2007), especially since an imbalance between fission and fusion appears to have a causal role in initiating several adult neurodegenerative diseases (Bossy-Wetzel et al., 2003; Wang et al., 2009). For example, in Parkinson’s and Alzheimer’s diseases, impaired fission/fusion can lead to an increase in a large mitochondrial pool, with a medium-sized population remaining relatively intact (Trimmer et al., 2000). Similar change has been observed in anesthesia-induced mitochondrial size distribution (Sanchez et al., 2011).

Although mitochondrial enlargement is being noted in very immature neurons, the existing literature often refers to large (and “giant”) mitochondria in aging neurons, where they seem to be a common occurrence (Navarro and Boveris, 2010). Could we draw a parallel between certain elements of mitochondrial neurodegeneration unique to aging and anesthesia-induced mitochondrial neurodegeneration unique to the developing brain? This should be further examined by determining how anesthesia affects developmental fusion and fission by focusing on various GTPase proteins (including Drp1, fis 1, OPA 1, and mitofusin 1 and 2) that are critical for proper fusion and fission pathway activation (Smirnova et al., 2001; Zorzano et al., 2010).

Since mitochondria are generated in the neuronal body, initial work was focused on the morphological appearance of mitochondria located at this site; however, it is of equal importance to understand how anesthesia-induced morphological changes affect mitochondrial migration since they have to move within the cytoplasm to distribute within cells (Yaffe, 1999). Neurons have multiple compartments (e.g., dendrites, axons, and synapses) that extend far from the cell body, thus they depend heavily on proper mitochondrial distribution (Morris and Hollenbeck, 1993). As the main regulators of ATP production, mitochondria frequently are found in the vicinity of active growth cones of developing neurons (Morris and Hollenbeck, 1993) and in terminals with active synapses (Shepherd and Harris, 1998; Rowland et al., 2000). We have suggested that significantly fewer mitochondria are located in presynaptic neuronal profiles in anesthesia-treated brain than are in controls (Sanchez et al., 2011). Since the mitochondria also are significantly enlarged, it was proposed that anesthesia causes mitochondria to be sluggish and “stuck” in more proximal cellular compartments, thus shifting the regional distribution of mitochondria away from very distant, thin, and highly arborized dendritic branches at a time when their presence is necessary for normal synapse formation and development. In fact, we and others have reported that anesthesia impairs plasticity of dendritic spines and the formation, stability, and function of developing synapses (Head et al., 2009; Briner et al., 2010, 2011; Lunardi et al., 2010). For example, Briner et al. (2011) found that propofol decreased synaptic spine density in P5 rat medial prefrontal cortex, but increased spine density in P15–P25 rats. Briner et al. (2010) also found that isoflurane, sevoflurane, or desflurane affected synaptogenesis (increased dendritic spine density) in P16 rats in the medial prefrontal cortex, but no cell death.

What are the consequences of mitochondrial impairment? The morphological distortion and impairment of mitochondrial regional distribution is accompanied by production of excessive reactive oxygen species (ROS) and significant peroxidation of cellular and subcellular lipid membranes (Halliwell, 1992). This is important in the development and progression of several neuronal diseases that are marked by severe cognitive decline (Trushina et al., 2004; Bennett, 2005; Reddy, 2006, 2007). Neurons are highly dependent on glucose for ATP synthesis and produce ROS as byproducts of oxidative phosphorylation in mitochondria. Because of their high oxygen requirements and relative deficiency in oxidative defenses – in particular, low to moderate activity of catalase and Mn-superoxide dismutase (SOD) – neurons are highly sensitive to excessive ROS production. This vulnerability, combined with their high content of polyunsaturated fatty acids, makes them susceptible to excessive lipid peroxidation and cellular damage (Halliwell, 1992). Indeed, early exposure to anesthesia makes developing neurons susceptible to ROS up-regulation, mitochondria-induced ROS-propagated lipid peroxidation, and neuronal deletion that may contribute to the observed impairment of cognitive development. Our recent study has hinted a functional link between the disturbances in mitochondrial function and ROS up-regulation and cognitive disturbances. EUK-134, a synthetic ROS scavenger having both Mn-SOD and catalase activity (Baker et al., 1998; Liu et al., 2003), or R(+) pramipexole [R(+) PPX], a synthetic aminobenzothiazole derivative that blocks permeability transition pores, restore the integrity of mitochondrial membranes (Sayeed et al., 2006), and limit ROS production (Cassarino et al., 1998; Le et al., 2000; Zou et al., 2000). By curtailing ROS up-regulation and lipid peroxidation, EUK-134 and R(+) PPX not only preserve mitochondrial morphogenesis and neuronal viability, but also prevent the cognitive impairment in adolescent rats that were exposed to GAs during synaptogenesis (at post-natal day 7). This suggests that anesthesia neurotoxicity is the result of a highly complex interaction between mitochondria-induced and ROS-propagated cascades of events that ultimately leads to neuronal damage and behavioral impairment. Thus, preventing excessive lipid peroxidation and protecting mitochondria could be promising strategy for safe use of GAs during early stages of brain development.

Other protective strategies based on preserving mitochondrial integrity have been shown previously to provide significant inhibition of anesthesia neurotoxicity. For example, melatonin, a naturally occurring sleep hormone that upregulates bcl-x_L_ (Yon et al., 2006) and prevents cytochrome *c* leak, and carnitine, a nutritional supplement that protects mitochondrial integrity (Zou et al., 2008), cause significant protection against neuronal apoptosis. Although it remains to be determined whether melatonin and carnitine also protect against anesthesia-induced cognitive impairment, it is clear that mitochondria play an integral role in proper development of the immature neurons and their synaptic connections.

## The Role of Endoplasmic Reticulum in Anesthesia-Induced Developmental Neurotoxicity

In considering the described impairment of mitochondrial function and the downstream consequences, we must keep in mind that the upstream trigger could be the excessive release of calcium from the ER resulting in cytosolic and mitochondrial calcium overload. This, in turn, may cause cytochrome *c* leak (Hanson et al., 2004), which could further promote mitochondrial dysfunction (Figure 1). The ER could be an important initial target of anesthesia neurotoxicity. Indeed, Zhao et al. (2010) have shown that by activating inositol 1,4,5-trisphosphate receptors, the inhalational anesthetic isoflurane induces significant calcium release from the ER resulting in acute elevation of cytosolic calcium and modulation of mitochondrial bcl-x_L_ protein, which then promotes apoptotic neuronal death in the immature rat brain. Similar modulation of inositol 1,4,5-trisphosphate receptors was reported with GAs propofol, desflurane, and sevoflurane, with resultant cytosolic calcium overload and an increase in mitochondrial permeability transition pore activity (Inan and Wei, 2010). This increased pore activity was shown to cause mitochondrial swelling, resulting in uncontrolled release of pro-apoptotic factors.

Although a moderate increase in calcium release via the activation of inositol 1,4,5-trisphosphate receptors may provide neuroprotection in some forms of brain injury (Wei et al., 2007), excessive activation of these receptors may lead to elevation of intracellular calcium into the toxic range. As a second messenger, intracellular calcium regulates many aspects of neuronal development including synapse development and functioning, membrane excitability, protein synthesis, neuronal apoptosis, and autophagy – in short, all important elements of neuronal survival (Hanson et al., 2004; Berridge, 2009; Decuypere et al., 2011). Disturbance in calcium homeostasis has been considered responsible for some forms of learning and memory deficits (Power et al., 2002; Rosenzweig and Barnes, 2003). Since the ER is the primary source of releasable calcium in neurons, it plays an important role in neuronal function and survival.

## The Role of Lysosomes in Anesthesia-Induced Developmental Neurotoxicity

Since damaged mitochondria could become an uncontrollable source of free oxygen radicals and a damaged ER could become an uncontrollable source of intracellular calcium, the concern is that anesthesia may produce a substantial amount of defective organelles, often referred to as toxic biological “garbage,” that have to be degraded to ensure neuronal survival. Large organelles can be cleared without causing substantial damage to the neurons by autophagy. Autophagy, a multistep process, is initiated by the formation of autophagosomes that enter lysosomes, the acidic vacuolar compartment (Brunk and Terman, 2002; Levine and Yuan, 2005; Terman et al., 2007). Defective organelles are autophagocytized at a low rate, resulting in lysosomal accumulation of an undegradable, polymeric, autofluorescent material called lipofuscin.

It appears that GA heightens the formation of autophagic bodies (Sanchez et al., 2011) (Figure 1). For example, after anesthesia, pyramidal neurons in developing subiculum, a part of hippocampus that is exquisitely sensitive to anesthesia neurotoxicity, showed a substantial number of apoptotic profiles containing autophagosomes, lysosomes, and autophagic vacuoles. The increase in the autophagic load raises the possibility that anesthesia kills developing neurons by inducing “autophagic stress” – by overwhelming natural autophagy due to a massive production of defective organelles, by ramping up lysosomal activity, or by some combination of both. A relationship between autophagy and neuroapoptosis has been suggested in other models of cell injury. Some argue that autophagy is critical for the initiation of apoptosis (Orrenius et al., 2011), while others suggest that autophagy and apoptosis are independent of each other (Gozuacik and Kimchi, 2004).

It is possible that anesthesia-induced autophagy is a defense mechanism aimed at clearing damaged mitochondria although it could also be the outcome of derangements in intracellular calcium signaling due to anesthesia-induced ER dysfunction. For example, abnormal calcium release from the ER via inositol 1,4,5-trisphosphate receptors has been shown to activate autophagy, and it seems to occur via activation of the mammalian target of rapamycin (mTOR) part of an atypical serine/threonine kinase-dependent pathway (Hoyer-Hansen et al., 2007). Although it is unknown whether GAs play a role in the activation of this pathway, it can be argued that anesthesia-induced “self-eating” may be due to impaired function of lysosomes. The calcium concentration in lysosomes, which is regulated by nicotinic acid dinucleotide phosphate (NAADP)-gated two-pore channels (TPCs), is important for endosomal-lysosomal trafficking (Zhu et al., 2010). Although the scientific focus has been on diseases of impaired, not enhanced autophagy, it is possible that, by causing mitochondrial damage, ROS up-regulation, and lipid peroxidation, GAs induce more direct disturbance of lysosomal function with high calcium levels that cause overactivation of endosomal-lysosomal trafficking.

## Potential Clinical Importance of Research Findings

Although the basic science evidence regarding the deleterious effects of GAs on cognitive and behavioral development is very compelling, the clinical relevance remains to be established. Nevertheless, recently emerging retrospective human studies suggest that early exposure to GAs may have deleterious effects on human psychological, emotional, and cognitive development leading to learning deficits and hyperactivity disorders later in life (Hack et al., 2005; Hintz et al., 2005; Chorne et al., 2007; Rees et al., 2007; Sun et al., 2008; Kalkman et al., 2009; Wilder et al., 2009; Sprung et al., 2012).

It appears that the timing and duration of anesthesia exposure are important. Very young children sedated in intensive care units for days (e.g., for status epilepticus or asthmaticus) may be at a very high risk, especially if they are exposed during intense synaptogenesis when the effects of GAs seem to be most detrimental for neuronal survival and/or proper synapse formation. If, indeed, the developing human brain is vulnerable to the apoptotic cell death and impairment of synaptogenes is observed in developing animal brains, it is of great importance to know, as accurately as possible, when synaptogenesis occurs in the developing human brain. The last trimester of *in utero* life and the first few years of post-natal life could be most crucial. In fact, recent studies have reported regressive behavioral changes in children exposed to GAs before the age of 4 years, suggesting that this could be the most vulnerable age group (Sun et al., 2008; Wilder et al., 2009).

It is our moral obligation to pursue a better understanding of poor neurocognitive outcomes that could be anesthesia-induced. The fact that we are able to carry the sickest children through the most critical phases of their diseases and to keep them alive should not lull us into believing that our work is done. We have to continue our efforts to provide improved care that will not have long-term devastating effects on the patients’ cognitive and behavioral well-being. Thus, we must improve our understanding of the mechanisms that underlie the neurotoxicity of GAs so that preventive strategies could be developed especially in cases when life-threatening conditions make frequent anesthesia exposures a necessity that cannot be avoided.

## Conflict of Interest Statement

The authors declare that the research was conducted in the absence of any commercial or financial relationships that could be construed as a potential conflict of interest.
